# An Unusual Cause of Severe Pulmonary Hypertension: Compression of Pulmonary Arteries by Aortic Aneurysm and Thrombus Formation

**DOI:** 10.7759/cureus.90653

**Published:** 2025-08-21

**Authors:** Ramla Warsame, Ruth Ofuya, Ruju Smirtha Thillai, Uday Gollapinni, Olufunbi Kupoluyi, Ahmed Malik Abuelgasim Malik, Sadia Sultana

**Affiliations:** 1 Acute Internal Medicine, University Hospital of North Midland, Royal Stoke University Hospital, Stoke-on-Trent, GBR; 2 Acute Medicine, University Hospital of North Midland, Royal Stoke University Hospital, Stoke-on-Trent, GBR; 3 Acute Medicine, Royal Stoke University Hospital, Stoke-on-Trent, GBR

**Keywords:** aortic aneurysm, ct pulmonary angiography, intraluminal thrombus, non-operative management, pulmonary hypertension, right heart failure, right main pulmonary artery, thoracic aortic aneurysm (taa), vascular compression, virchow’s triad

## Abstract

Pulmonary hypertension (PHTN) has a broad differential, ranging from common aetiologies like thromboembolism, chronic lung disease, and heart failure. External compression of the pulmonary arteries by thoracic aortic aneurysms (TAAs) is an exceptionally rare cause of PH but may significantly impact haemodynamics. We report the case of an 81-year-old woman presenting with progressive dyspnoea and peripheral oedema, who was found to have near-complete compression of the right main pulmonary artery (RMPA) by an ascending aortic aneurysm, along with intraluminal thrombus. Transthoracic echocardiography revealed severe PH with preserved left ventricular (LV) function. CT pulmonary angiography was carried out, confirming both mechanical compression of RMPA and thrombus formation. According to the existing literature, we proposed that compression-induced stasis likely contributed to thrombosis, aligning with Virchow’s triad. Given the patient's frailty and comorbidities, she was managed conservatively. The multifactorial nature of this case prompts us to consider broader differentials in unexplained PH. It also acknowledges the challenges in managing larger symptomatic aneurysms in the elderly population.

## Introduction

Pulmonary hypertension (PHTN), defined as a mean pulmonary arterial pressure (mPAP) greater than 20 mmHg, can result from left heart disease, chronic lung disease, and thromboembolism [1]. While pulmonary embolism is a recognised cause, rare mechanical causes such as external compression of the pulmonary arteries may also lead to PH but are infrequently reported. Thoracic aortic aneurysms (TAAs) are typically asymptomatic and only rarely associated with heart failure. However, due to their close anatomical relationship to the pulmonary arteries, they can exert a significant mass effect when sufficiently enlarged, increasing pulmonary vascular resistance and leading to right ventricular dysfunction [1,2]. This phenomenon remains scarcely reported in literature, with only a few documented cases demonstrating significant PHTN as a result of pulmonary artery compression by aortic aneurysms; however, coexisting pulmonary thrombosis and vascular compression are exceptionally rare and pose a treatment challenge [3].

We report the case of an 81-year-old woman who presented with progressive dyspnoea and was found to have an ascending aortic aneurysm causing near-complete compression of the right main pulmonary artery. This resulted in severe PHTN and right heart failure [4]. The case highlights the importance of considering vascular compression in the differential diagnosis of unexplained or disproportionate PHTN, particularly in patients with known aortic aneurysmal disease. 

## Case presentation

An 81-year-old woman was referred to acute medical services with progressive dyspnoea, cyanosis, and bilateral lower limb oedema. Her symptoms had worsened gradually over several weeks, and she had independently commenced 1 L/min home oxygen therapy. A recent outpatient computed tomography (CT) thorax arranged by her general practitioner to investigate suspected interstitial lung disease (ILD) demonstrated interval enlargement of her known ascending aortic aneurysm from 58 mm to 67 mm.

Her medical history includes heart failure with preserved left ventricular ejection fraction (49%), PHTN, atrial fibrillation on apixaban, hypertension, and prior breast cancer. She had an aortic root surgery in 2014, and follow-up computed tomography (CT) of the chest showed the development of a pseudo-aneurysm in her ascending aorta. She has since been under the cardiothoracic team for surveillance and has been deemed unsuitable for surgical re-intervention due to comorbidities, frailty, and risk of operative mortality.

**Figure 1:**
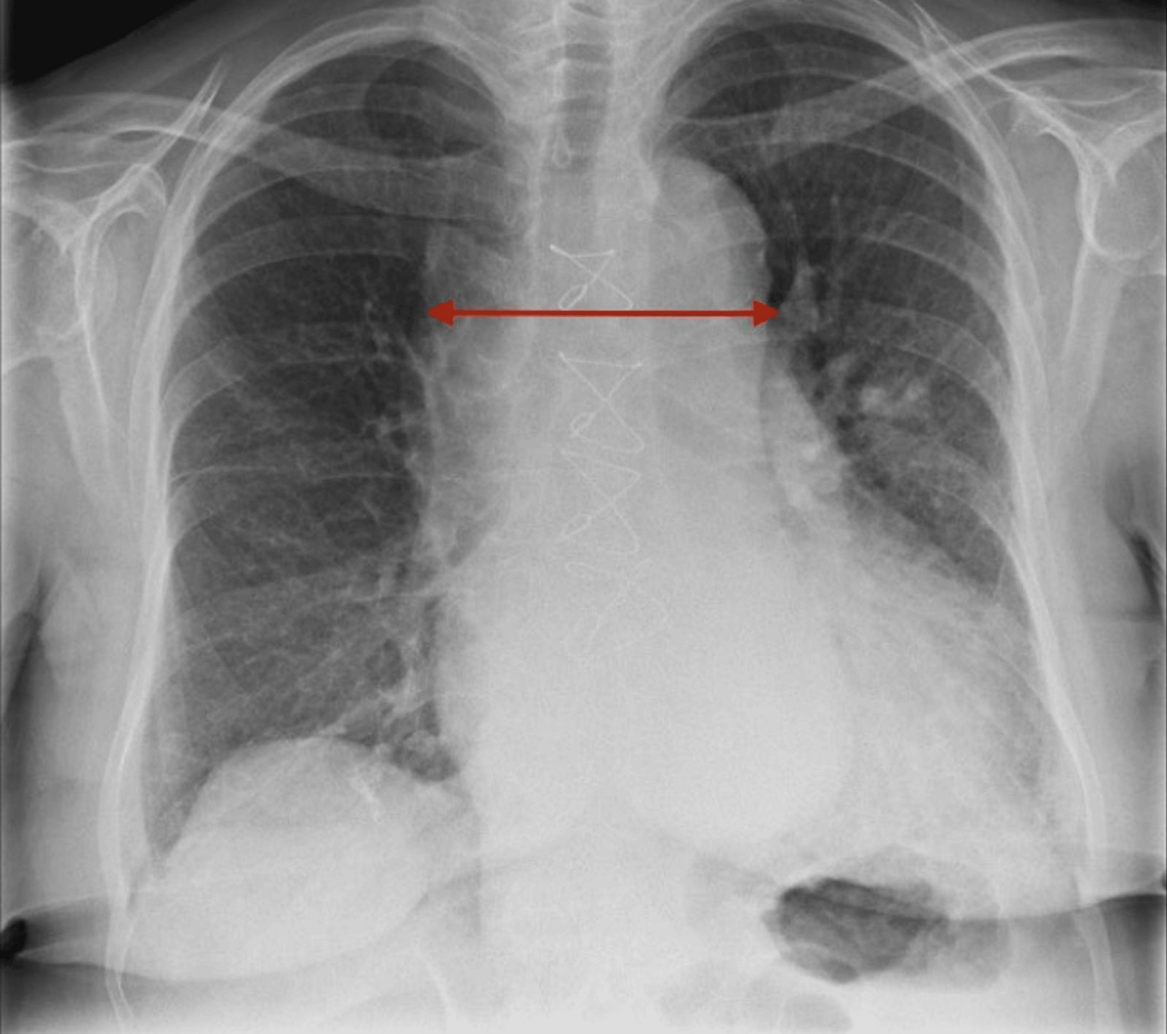
Chest X-ray (CXR) The red line on the CXR shows the widened mediastinum.

On examination, she was tachypneic with a respiratory rate (RR) of 23, maintaining oxygen saturations at 95% on room air. Bibasal crackles and oedema up to the knees were noted. Arterial blood gas showed type 1 respiratory failure, routine bloods revealed polycythaemia, and a D-dimer of 3664 ng/mL, with normal inflammatory and renal markers. The chest radiograph had no significant pulmonary congestion or volume overload (Figure 1).

A bedside echocardiogram demonstrated a dilated right atrium and ventricle, severe PHTN, preserved left ventricular systolic function, and a dilated aortic root. These findings were consistent with a prior outpatient echocardiogram, which had shown pulmonary pressures out of proportion to left heart findings. The case had also been discussed at the multidisciplinary heart failure meeting two weeks before admission, where it was agreed that left heart failure was unlikely to account for her symptoms, and further investigation was advised.

**Figure 2:**
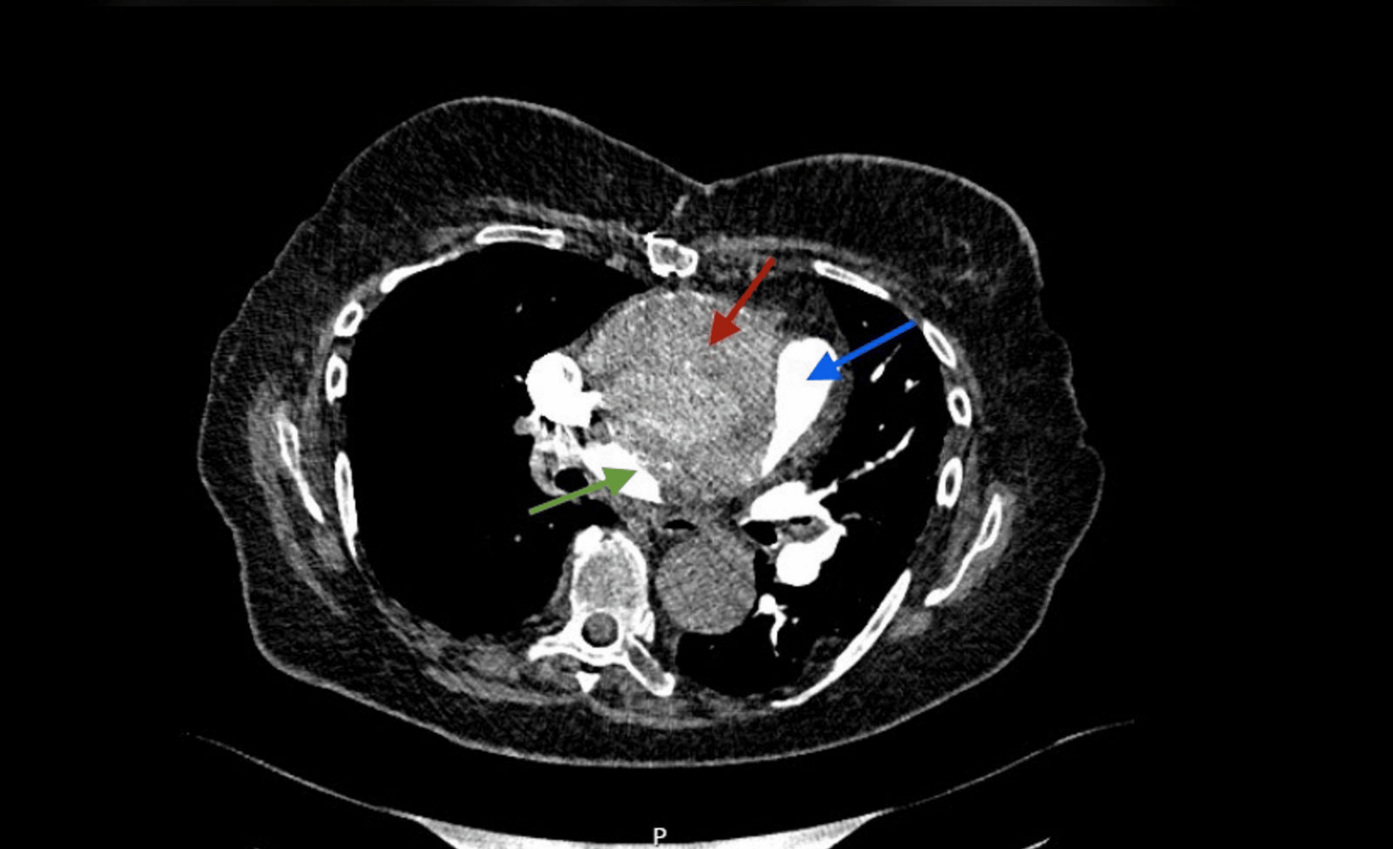
Computed tomography pulmonary angiogram (CTPA) CTPA showing an increase in size of the pseudo-aneurysm ventrally. The diameter of the ascending aorta has increased to 67 mm (was 58 mm). There is a thrombus in the pulmonary trunk and the right main pulmonary artery, resulting in marked narrowing of the residual lumen. The red arrow shows the aneurismatic ascending aorta. The blue arrow shows the pulmonary trunk. The green arrow shows the right main pulmonary artery with thrombus.

The initial working diagnosis was PHTN of unclear mechanism, possibly Group 3 or 4. Respiratory input was sought following admission, where they reviewed all existing computed tomography (CT) imaging in a multidisciplinary thoracic radiology meeting and excluded significant interstitial lung disease. Instead, the CT pulmonary angiogram (CTPA) demonstrated interval enlargement of the ascending aortic aneurysm to 67 mm, with near-complete external compression of the right main pulmonary artery. In addition, thrombus was seen within the pulmonary trunk and right pulmonary artery (Figure 2). These findings confirmed the multifactorial cause of the disease.

Anticoagulation was escalated to treatment-dose dalteparin, and intravenous furosemide was initiated. Cardiothoracic surgery, already involved in her follow-up, advised continuing conservative management in view of her age and comorbidity. Interventional radiology was consulted but deemed endovascular options unsuitable due to anatomical constraints and reduced physiologic reserve. She was later transitioned to palliative care following clinical deterioration.

Below is the table of the blood results (Table 1).

**Table 1:** Blood test results Hb: hemoglobin, Hct: hematocrit, WBC: white blood cell count, PLT: platelet count, INR: International Normalized Ratio, Na: sodium, K: potassium, eGFR: estimated glomerular filtration rate, CRP: C-reactive protein, Mg: magnesium, Ca: calcium, ALT: alanine aminotransferase

Investigation	Result	Normal reference range
Hb	165	115–165 g/L
Hct	0.49	0.36–0.47 L/L
WBC	5.8	4–11 x 10⁹/L
PLT	164	150–450 x 10⁹/L
INR	1.8	0.8–1.2 (if not on anti-coag)
Na	140	133–146 mmol/L
K	4.5	3.5–5.3 mmol/L
Urea	5.2	2.5–7.8 mmol/L
Creatinine	57	45–84 mmol/L
eGFR	83	>90 mL/min/1.73m²
CRP	<4	0–5 mg/L
Phosphate	1.22	0.8–1.5 mmol/L
Mg	0.91	0.70–1.0 mmol/L
Ca	2.58	2.2–2.6 mmol/L
ALT	13	0–34 IU/L
Total bilirubin	21	0–21 µmol/L
D-dimer	3664	<500 ng/mL

## Discussion

Mechanical compression of the pulmonary arteries is an uncommon but important cause of PHTN. The proximity of the ascending aorta to the pulmonary artery bifurcation makes the right pulmonary artery particularly susceptible to mass effect in cases of large aneurysms. In such scenarios, reduced pulmonary blood flow increases vascular resistance and results in right-sided strain [1,2].

In this case, CTPA identified both near-complete compression of the RMPA and intraluminal thrombus. Although either mechanism can independently cause PH, their combination is rarely reported. Most published cases describe isolated aneurysmal compression of the pulmonary arteries without associated thrombosis or dissection [1,3], whereas others involve dissecting or ruptured aneurysms causing compression through mass effect from an adjacent hematoma [2,4].

This combination may also mimic isolated pulmonary embolism, with similar clinical and radiographic findings, previously described in the literature, where compression from an aortic aneurysm was misinterpreted as pulmonary embolism on imaging [5]. In our patient, disproportionate right heart strain with preserved left heart function prompted CTPA, which ultimately confirmed the dual pathology.

The pathophysiology of this dual pathology can best be explained by Virchow’s triad of stasis, endothelial injury, and hypercoagulability. The expanding aneurysm likely impaired blood flow through the constricted part of the pulmonary artery and induced localised stasis, promoting in situ thrombosis. This mechanism has been previously reported, where a large unruptured abdominal aortic aneurysm (AAA) led to pulmonary embolism through compression of the inferior vena cava, causing venous stasis and subsequent thrombosis [6].

In our case, the aneurysm may have both obstructed pulmonary flow, creating a favourable environment for thrombosis, which further contributes to the observed severity of PHTN.

Management in such cases is widely based on anatomical proximity and physiological reserve. In surgically fit patients, repair of the aneurysm may relieve vascular compression and improve haemodynamics [2], either through open or endovascular means. However, in frail individuals with multiple comorbidities, the risks of intervention often outweigh potential benefits. Our patient had been under cardiothoracic surveillance and was previously deemed unsuitable for operative repair. This case underscores the importance of evaluating for mechanical contributors in patients with unexplained or disproportionate PH. Dual pathology may be under-recognised and contributes to rapid haemodynamic compromise and poor prognosis. CTPA remains the modality of choice for simultaneously assessing thrombotic and structural pathology.

## Conclusions

This case illustrates a rare and diagnostically complex presentation of severe PHTN resulting from external compression of the right main pulmonary artery by an enlarging ascending aortic aneurysm, a delayed complication of prior aortic root surgery. Progressive aneurysmal enlargement led to significant vascular obstruction, right heart strain, and respiratory failure, further complicated by intraluminal thrombus formation.

This report underscores the importance of recognising uncommon mechanical causes of PHTN, particularly when clinical findings appear disproportionate to more common aetiologies. While TAAs are often asymptomatic, they may exert a significant mass effect on adjacent pulmonary arteries, leading to haemodynamic compromise. Such compression can not only obstruct flow but also promote localised stasis, predisposing to thrombosis. When thrombotic burden alone does not fully explain the severity of PHTN, structural compression should be considered. CTPA remains the key imaging modality for detecting both thrombotic and anatomical causes. In frail or non-operative candidates, conservative management is often the only feasible approach.
